# Oral nicotine pouches with an aftertaste? Part 1: screening and initial toxicological assessment of flavorings and other ingredients

**DOI:** 10.1007/s00204-023-03538-9

**Published:** 2023-06-30

**Authors:** Nadja Mallock-Ohnesorg, Selina Rinaldi, Sebastian Malke, Nadine Dreiack, Elke Pieper, Peter Laux, Thomas Schulz, Ralf Zimmermann, Andreas Luch

**Affiliations:** 1 Department of Chemical and Product Safety, German Federal Institute for Risk Assessment (BfR), Berlin, Germany; 2 Chair of Analytical Chemistry, Joint Mass Spectrometry Centre, University of Rostock, 18059 Rostock, Germany

**Keywords:** Nicotine pouches, Aroma screening, Untargeted analysis, Toxicological assessment

## Abstract

Nicotine pouches are oral products that deliver nicotine without containing tobacco. Previous studies mainly focused on the determination of known tobacco toxicants, while yet no untargeted analysis has been published on unknown constituents, possibly contributing to toxicity. Furthermore, additives might enhance product attractiveness. We therefore performed an aroma screening with 48 different nicotine-containing and two nicotine-free pouches using gas chromatography coupled to mass spectrometry, following acidic and basic liquid–liquid extraction. For toxicological assessment of identified substances, European and international classifications for chemical and food safety were consulted. Further, ingredients listed on product packages were counted and grouped by function. Most abundant ingredients comprised sweeteners, aroma substances, humectants, fillers, and acidity regulators. 186 substances were identified. For some substances, acceptable daily intake limits set by European Food Safety Agency (EFSA) and Joint FAO/WHO Expert Committee on Food Additives are likely exceeded by moderate pouch consumption. Eight hazardous substances are classified according to the European CLP regulation. Thirteen substances were not authorized as food flavorings by EFSA, among them impurities such as myosmine and ledol. Three substances were classified by International Agency for Research on Cancer as possibly carcinogenic to humans. The two nicotine-free pouches contain pharmacologically active ingredients such as ashwagandha extract and caffeine. The presence of potentially harmful substances may point to the need for regulation of additives in nicotine-containing and nicotine-free pouches that could be based on provisions for food additives. For sure, additives may not pretend positive health effects in case the product is used.

**Supplementary Information:**

The online version contains supplementary material available at 10.1007/s00204-023-03538-9.

## Introduction

New nicotine-releasing products, all-white nicotine pouches, have been introduced to the market in 2016 in the US and 2018 in Europe, respectively (Delnevo et al. 2021). These products are small pouches that are filled with powder. As no tobacco leaf material is present in the final product, nicotine has been added to the cellulose-based powder alongside other ingredients (Robichaud et al. 2020). For consumption, the pouches are placed between upper lip and gum where they remain for several minutes. Consequently, substances released from the pouches could locally affect the oral mucosa but may also act systemically upon resorption. Previous studies have focused on the contents or release of nicotine and other known tobacco toxicants (Aldeek et al. 2021; Azzopardi et al. 2021; Mallock et al. 2022; Stanfill et al. 2021). Besides high nicotine contents of almost 50 mg per pouch, low levels of tobacco carcinogens such as *N*-nitrosonornicotine (NNN) or 4-(methylnitrosamino)-1-(3-pyridyl)-1-butanone (NNK) have been reported (Mallock et al. 2022). These studies on known tobacco toxicants can help to classify nicotine pouches among other nicotine delivery products within a potential harm minimization continuum (Abrams et al. 2018). However, for a comprehensive risk assessment of these products, a broader knowledge on their hazardous constituents is required. In particular, this refers to substances that are not typical for tobacco products and thus might have been missed by the above-mentioned studies. Using a more comprehensive approach, potentially hazardous substances present in nicotine pouches need to be identified and evaluated regarding their hazard potential.

**Fig. 1:**
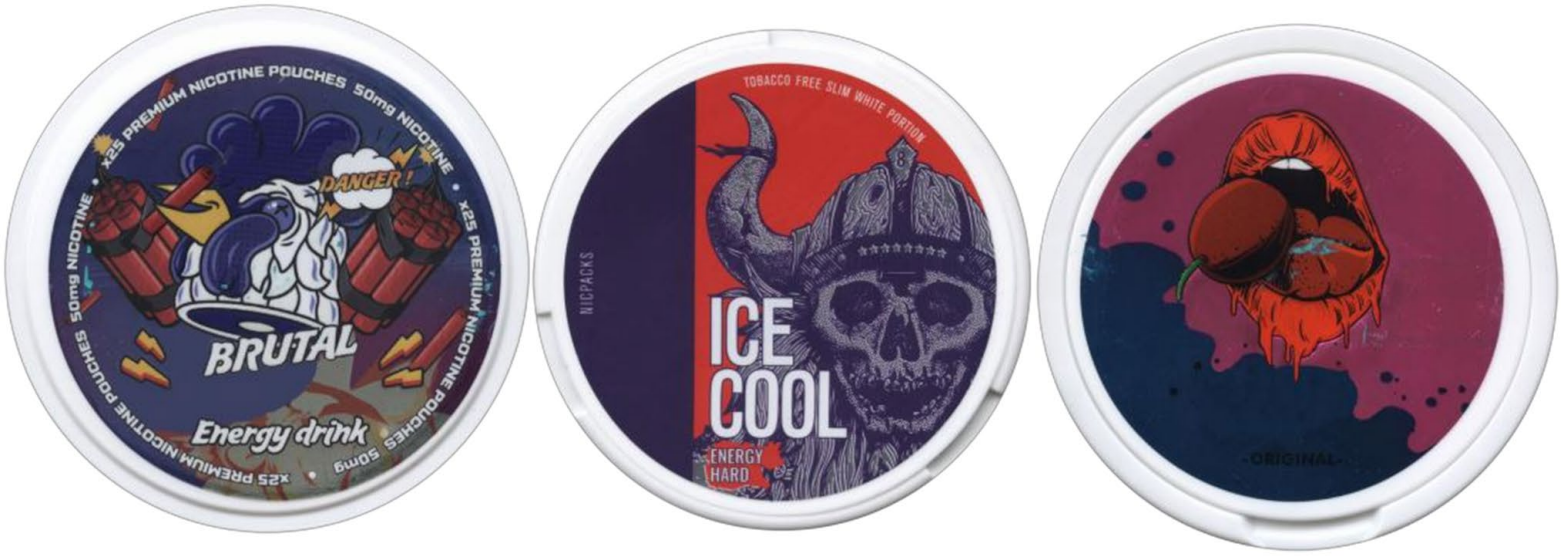
Some examples of nicotine pouch packages (brand names removed)

Besides their potential toxicological relevance, additives in tobacco and related products can enhance product attractiveness or addictiveness (SCENIHR 2010). It should be kept in mind that nicotine pouches can contain considerable amounts of nicotine (Mallock et al. 2022), a highly addictive substance (Henningfield and Keenan 1993). If such products are being picked up by previous non-nicotine users, the number of people addicted to nicotine is likely to increase. Individuals initially consuming nicotine pouches may continue product use or even start to smoke cigarettes. Therefore, they will be avoidably exposed to hazardous compounds resulting in an increased health risk at the individual level and a negative impact on public health in general (Lund and Vedoy 2021). Therefore, the health risk assessment of such products needs to consider additives that enhance product attractiveness or addictiveness. First, additives can enhance product addictiveness. For instance, nicotine uptake via the oral mucosa can be facilitated by pH modulators. The pH increase leads to a higher proportion of the free-base nicotine which can pass the oral mucosa more rapidly due to its nonpolar character (Pickworth et al. 2014; Tomar and Henningfield 1997). In a recent study, it was shown that the majority of analyzed nicotine pouches in fact elicited an alkaline pH value (Mallock et al. 2022). Second, additives, such as flavorings, sweeteners, or flavor enhancers, create tastes that are likely to attract novice users and the young (Hoffman et al. 2016). For instance, this has been discussed for snus, an oral tobacco product (Kostygina and Ling 2016). Likewise, nicotine pouches are sold with various flavors that might target young people in particular (Robichaud et al. 2020). Product packages with flashy designs as displayed in Fig. 1 add to this suspicion.

This study was performed to address open questions regarding potential health risks in two separate parts. For the part described herein, an untargeted screening of 48 different nicotine pouches and two pouches without nicotine was performed by applying gas chromatography and mass spectrometric detection. Substances were identified using commercial and in-house libraries. Subsequently, an initial toxicological assessment based on European and international provisions for chemical and food safety was performed for each compound identified. For comparison purposes, product packages were examined to get information on the flavors and ingredients declared. In the second part of this study, which is presented in another paper, in vitro toxicity was studied for five different nicotine pouches in human gingival fibroblasts (HGF-1) in vitro to look into potential local toxic effects (Rinaldi et al. 2023, submitted).

## Materials and methods

### Nicotine pouches

Forty-eight different packages with nicotine pouches and two packages containing pouches without nicotine from 22 different brands were bought at German online shops or at online shops located in foreign countries. Samples were selected to cover different flavors and nicotine contents.

### Chemicals and standard substances

All used solvents were of analytical or higher purity grade. Ethyl acetate, hydrochloric acid, nicotine, and ammonia were bought from Merck KGaA (Darmstadt, Germany). For standard substances, the source is stated in the section Supplementary Material. Ultra-pure water was prepared using a Milli-Q Integral Water Purification System (Merck KGaA, Darmstadt, Germany).

### Assessment of package for product flavors and listed ingredients

Product labels were examined regarding product flavors and listed ingredients. Ingredients were assessed by their frequency of appearance and grouped based on their potential function. Identified product flavors were noted and grouped into flavor categories based on a flavor wheel that has been proposed by Krüsemann et al. for e-liquid flavors (Krusemann et al. 2019). The frequency of the appearance of certain flavors was not recorded, since sample selection was not representative.

### Identification of flavorings used in nicotine pouches

Flavorings were identified using gas chromatography coupled to mass spectrometric detection (GC/MS) as described (Hutzler et al. 2014), with an adaption of the liquid–liquid-extraction (LLE) protocol. Nicotine pouches were placed in Erlenmeyer flasks to which 5 mL ultra-pure water were added. Products were extracted subsequently with 5 mL ethyl acetate under acidic conditions after addition of 5 mL 0.1 M hydrochloric acid and under basic conditions after addition of 5 mL 0.2 M ammonia, respectively. 2 µL of the organic phase were injected into a GC/MS system (Agilent 6890 and 8890 gas chromatograph, MSD 5975c mass spectrometer, Agilent Technologies, Waldbronn, Germany) equipped with a Multipurpose Sampler (MPS 2 XL) and a Cold Injection System (CIS) from Gerstel (Mühlheim an der Ruhr, Germany). Separation on a DB-17 ms capillary column (30 m × 0.25 mm I.D., 0.25 µm film thickness; Agilent Technologies, Waldbronn, Germany) and analysis was carried out as described (Hutzler et al. 2014). Peaks were identified using the software Mass Hunter Qualitative Analysis version 10.0 (Agilent Technologies, Waldbronn, Germany) and MSD ChemStation version F.01.03.2365 (Agilent Technologies, Waldbronn, Germany) and three different spectra libraries; NIST 11 spectral library, FFNSC 3, and an in-house aroma library created with standard substances analyzed together with nicotine using the same method (see Supplementary Material). For substances that were included in the in-house library, identification was verified according to its relative retention time (RRT) with nicotine as reference (± 0.05).

### Toxicological assessment of ingredients

For the toxicological assessment of detected ingredients in nicotine pouch extracts and nicotine-free pouch extracts, the following sources were used: The database compiled by the Joint FAO/WHO Expert Committee on Food Additives (JECFA) (World Health Organizaton 2021) and the food flavorings database provided by the European Commission based on the Annex I to Regulation (EC) No. 1334/2008 (European Parliament and the Council of the European Union 2008a). In addition to the food flavorings database, European Food Safety Agency (EFSA) opinions and recommendations on the respective flavors were considered. Further, the C&L Inventory by ECHA was searched for harmonized hazard classifications according to Regulation (EC) No 1272/2008 (CLP regulation) (European Parliament and the Council of the European Union 2008b). Finally, the substances were reviewed whether they are classified for carcinogenicity by the International Agency for Research on Cancer (IARC). A literature search was performed for substances with at least one of the following concerns; IARC classification, no authorization as food flavoring compound, and relevant hazard classification by CLP. It was checked, if the acute toxicity qualified for hazard class Acute Tox 1 or 2. Hazard classes Acute Tox 3 or 4 were considered irrelevant due to its high LD50 values. Skin irritation and corrosion as well as eye damage and irritation were considered irrelevant, since pouches are applied orally and substance concentrations are usually small. That leaves the following hazard statements, which are considered relevant for a toxicological assessment of nicotine pouch flavorings (H315, H317, H361d, and H372).

### Calculation of content limits based on the acceptable daily intake (ADI)

EFSA of JECFA allocated acceptable daily intake (ADIs) to certain substances. For these, content limits per pouch were calculated for two scenarios. To consider an “average scenario”, it was assumed that a person with a weight of 70 kg consumes five pouches per day. For the “worst-case scenario”, it was assumed that a person of 70 kg weight consumes 20 pouches per day. Both assumptions are based on previously published surveys (Havermans et al. 2021; Prasad et al. 2022). Since consumers are exposed to additives via serval exposure routes (e.g., food, cosmetics, and nicotine pouches), it was decided to calculate with 50% of the respective ADI value.

## Results

### Ingredients and flavors found on the declaration label of product packages

Lists of ingredients were found on the packages of 49 of the products investigated. One sample was a free sample that was sent without being ordered. On the package of this sample, the ingredients and other information were not disclosed at all. The ingredients listed on the other samples were counted and grouped into categories according to their potential function. The results are displayed in Table 1. Although some ingredients may have more than one potential function, each additive was grouped into only one category. For example, maltitol may have been added to function as a sweetener or as a filler. Further, more than one ingredient of a category may have been listed on one product. Most abundant ingredient categories with more than 40 counts were sweeteners, aroma additives, humectants (mostly propylene glycol), fillers (mostly cellulose and plant fibers), acidity regulators (mostly sodium carbonate), nicotine, and water. The two nicotine-free pouches contained several herbs containing active compounds, such as caffeine or ashwagandha.

**Table 1:** Listed ingredients that were counted on packages of 49 products, grouped into categories

Ingredient category	Total counts	Ingredients, counts
Sweeteners	57	Xylitol (E967), 17; acesulfame K (E950), 15; sucralose (E955), 15; erythritol (E968), 5; stevia (E960), 3; maltitol (E965), 2
Aroma	56	Undefined aroma or food flavors, 46; salmiac (E510), 6; mint aroma or corn mint oil, 3; L-menthone, 1
Humectants	54	Propylene glycol (E1520), 25; sodium alginate (E401), 11; glycerol (E422), 10; undefined humectant, 7; agar agar (E406), 1
Fillers	53	Cellulose (E460) and plant fibers, 37; fibers from eucalyptus and pine tree, 9; undefined fibers, 5; undefined fillers, 2
Acidity regulators	51	Sodium carbonate (E500), 34; undefined acidity regulators, 8; potassium carbonate (E501), 4; magnesium carbonate (504), 3; calcium lactate (E327), 1; citric acid (E330), 1
Nicotine	47	
Water	41	
Salt	27	Undefined salt, 25; Himalayan rock salt, 1; flavoring salt, 1
Thickener	19	Undefined gelling agent, 9; xanthan gum (E415), 7; guar gum (E412), 2; natural chewing gum basis, 1
Preservative	17	Potassium sorbate (E202), 11; undefined preservative, 6
Herbs^a^	12	Black tea, 2; green tea, 2; matcha, 2; ashwagandha, 2; herbal mixture, 2; guarana, 2
Flavor enhancer	7	Undefined flavor enhancer, 6; β-Cyclodextrine (E459), 1
Stabilizer	5	Hydroxypropyl cellulose (E463), 4; gum arabic (E414), 1
Other	4	Vegetable oil, 4

^a^Herbs were only listed on the two nicotine-free products

Product names were examined to identify the flavor. Flavors that could be identified are summarized in Table 2, grouped into flavor categories as previously proposed for e-liquids (Krusemann et al. 2019). Some products contained combinations of several flavors. Besides the flavor, 24 product names also indicated a cooling sensation effect of the product using the terms “cold”, “cool”, “eucalyptus”, “freeze”, “fresh”, “frost”, “ice”, “mint”, “polar”, “spearmint”, or “winter chill”. It should be noted that the list of used flavors is not exhaustive.

**Table 2:** Product flavors that were identified based on product names, grouped into flavor categories according to Krüsemann et al. (2019)

Flavor category as proposed by Krüsemann et al. (2019)	Identified flavors
Tobacco	Smokey, tobacco,
Menthol	Mint, spearmint
Spices	Chili, jalapeno, licorice, pepper
Coffee/tea	Black tea
Other beverages	Cola, energy drink
Fruit	Banana, berry, cassis, lemon, lime, melon, peach
Candy	Candy
Other sweets	Vanilla
Other	Eucalyptus

### Identified ingredients and toxicological assessment

Besides nicotine, a total of 186 different chemicals were identified in the 48 nicotine pouch extracts and 2 nicotine-free pouch extracts. A table presenting the identified substances per sample is provided in the Supplementary Material. The minimum and maximum numbers of substances per pouch were 4 and 32, respectively. A mean of 17 substances was found in each pouch. The complete list of identified compounds is also presented in the Supplementary Material. In Table 3, only compounds with either three or more hits or with a toxicological remark (e.g., ADI or hazard statement) are compiled. An in-house aroma compound library was created by measuring standard substances in the presence of nicotine with the same GC/MS method. Retention times and relative retention times in relation to nicotine are presented in the Supplementary Material.

A first toxicological assessment using the food flavorings database by the European Commission, the databases of JECFA, IARC, and CLP, revealed 47 substances having at least one concern according to one of these databases. Three substances are classified as possibly carcinogenic by IARC. In total, 13 substances do not have an authorization in the EU to be used as flavors. A hazard classification according to CLP was allocated to eight substances.

#### IARC classifications

Substances classified as possibly carcinogenic to humans (cat. 2B) by IARC were limited to methyl eugenol, benzophenone, and β-myrcene. All three substances were detected in less than four products.

Methyl eugenol induces liver tumors in rodents. The induction of cancer is based on a metabolic shift, meaning that only at high concentrations, the carcinogenic metabolite is formed by 1′-hydroxylation (Robison and Barr 2006). As the expected exposure levels are far below the ones used in the rodent carcinogenicity studies, the relevance for humans remains unclear (Smith et al. 2002). Based on the observed carcinogenicity in rodents and in vitro genotoxicity, methyl eugenol is not authorized on the European market for its use as a flavor additive (Scientific Committee on Food (SCF) 2001).

Benzophenone induces kidney and liver cancer as well as histiocytic sarcomas in rodents, thus being classified by IARC as possibly carcinogenic to humans (IARC Monographs Working Group on the Evaluation of Carcinogenic Risks to Humans 2012). EFSA, however, argues that there is no genotoxic effect observed, but has set a TDI of 0.03 mg/kg bw/day as a safe threshold (EFSA Panel on Food Contact Materials et al. 2017b).β-Myrcene is classified as possibly carcinogenic to humans by IARC based on renal and liver tumors found in rats and mice, respectively (IARC Monographs Working Group on the Evaluation of Carcinogenic Risks to Humans 2019). Regulatory bodies have not claimed safety concerns for humans at the levels of dietary intake. Although benzophenone and β-myrcene are authorized flavor additives in the EU, it still can be questioned whether it is permissible to add these substances of concern to lifestyle products such as nicotine pouches.

#### Substances not mentioned on the list of authorized food flavorings by the EU

Thirteen substances found in analyzed samples were not on the list of authorized flavorings: tris(2-butoxyethyl)phosphate, isomenthyl acetate, cis-β-farnesene, myosmine, ledol, saccharin, pulegone, isomenthol, neoisomenthol, humulene, cis-carvon oxide, estragole, and methyl eugenol.

Six among these substances (i.e., tris(2-butoxyethyl)phosphate, isomenthyl acetate, cis-β-farnesene, myosmine, ledol, and saccharin) are no flavoring substances. They could belong to the group of non-intentionally added substances (NIAS) or impurities. Myosmine, a tobacco alkaloid, is a known impurity of nicotine, either as a degradation product of nicotine or as a reagent used in nicotine synthesis (Jordt 2021; Wada et al. 1959). In fact, myosmine and minor tobacco alkaloids have been found in nicotine pouches in a previous study performed by a product manufacturer (Avagyan et al. 2021). Shortcomings in tobacco extract purification have become apparent in a recent study detecting tobacco-specific nitrosamines in extracts of some nicotine pouches (Mallock et al. 2022). Isomenthol, isomenthyl acetate, neoisomenthol, and pulegone have been present in a fair amount of samples but are not authorized as food flavorings. It is possible that these substances are impurities of mint extracts, since they can be found as ingredients of corn mint or peppermint oil (Boelens 1993; Tsai et al. 2013). Ledol is the main constituent of *Ledum palustre* (syn. *Rhododendron tomentosum, Ericaceae*)*.* This sesquiterpene has effects on the central nervous system, thereby causing dizziness, nausea, and vomiting, among other symptoms (Dampc and Luczkiewicz 2013). It can be also found in other plants, for instance, in oils of different eucalyptus variants (Anju et al. 2022; Gallon et al. 2020). Tris(2-butoxyethyl)phosphate is a flame retardant and plasticizer with possible hepatotoxic and carcinogenic properties (Saquib et al. 2022). It may originate from the pouch material and/or could also belong to the group of NIAS. Saccharin is a sweetening agent that does not fall under the Regulation (EC) No. 1334/2008.

For ashwagandha (“winter cherry”, *Withania somnifera*, nightshade family) that was listed on the packages of the two nicotine-free pouches, a hazard assessment based on the current literature was performed. Ashwagandha has been used as a medicinal plant for many years in India and is now increasingly popular in the western world as well. Mainly popular for its stress-relieving properties, it is also used for the treatment of several diseases, such as cancer, arthritis, cardiac and liver conditions etc., just to name a few. In a couple of studies, ashwagandha has been described as safe (Mandlik and Namdeo 2021). However, reports about drug-induced liver injuries in response to ashwagandha emerged recently (Philips et al. 2020). The reports were from Japan (Philips et al. 2020), USA, and Iceland (Bjornsson et al. 2020). A study by Siddiqui et al. showed that the phytochemical withanone, one of the substances that is found in high concentrations in ashwagandha, may play an important role due to its ability to form adducts with DNA and glutathione (GSH) (Siddiqui et al. 2021). The German Federal Institute for Risk Assessment (Bundesinstitut für Risikobewertung, BfR) has proposed to add ashwagandha to list C of Annex III of regulation (EC) No 1925/2006 on the addition of vitamins and minerals and of certain other substances to foods (“Substances under Community scrutiny”) (Klenow et al. 2013).

#### Classifications according to CLP

In total, eight substances were identified with a relevant hazard classification (H317, H361d, and H372): six (i.e., carvone, linalool, limonene, geraniol, isoeugenol, and citral) were classified as H317 (skin sensitizing properties). In addition, linalool, limonene, citral, geraniol, and isoeugenol are also listed as fragrance allergens according to Annex III of the Cosmetics Regulation (EC No. 1223/2009) (European Parliament and the Council of the European Union 2009). At air oxygen levels, linalool is quickly converted into a potent skin sensitizer via peroxidation (Skold et al. 2004). Since pouches are placed for several minutes into contact with the oral mucosa, most likely the mentioned compounds could also act as sensitizers locally in epithelial cells or even systemically upon absorption. Furthermore, the risk assessment committee (RAC) of the European Chemicals Agency (ECHA) classified salicylic acid into the hazard class Repr. 2 (H361d “suspected of damaging the unborn child”) (RAC 2019). Salicylic acid is also released from the human drug acetylsalicylic acid (ASA). RAC and the European Medicines Agency considered doses of up to 100 mg ASA/day as safe during pregnancy. This amount would correspond to 1.6 mg/kg bw/day of ASA for an individual of 60 kg (RAC 2016). Finally, benzoic acid has been categorized as STOT RE1 (H372 lung, inhalation), meaning that this compound “causes damage to the lung through prolonged or repeated exposure”. However, since benzoic acid in nicotine pouches is only applied orally, exposure via inhalation was considered irrelevant in this evaluation. Substances might have more than one classification.

### Content limits of substances with an ADI/TDI

EFSA derived ADI values for two substances and assigned a TDI value of 0.03 mg/kg bw/day to benzophenone. In addition, JECFA derived ADI values for 28 substances. These ADI values ranged from 0.1 to 25 mg/kg bw/day (Table 3). Considering two different ways of consumption, content limits in mg compound per pouch were calculated for these substances (Table 4). For benzophenone, contents of 0.21 and 0.05 mg/pouch would lead to an exceedance of the TDI in this scenario when 5 or 20 pouches were consumed per day, respectively.

**Table 3:** Compounds identified in 48 nicotine pouches and 2 nicotine-free pouches after liquid–liquid extraction and GC/MS analysis sorted by its frequency of occurrence

Substance	CAS	Number of hits	Annex I to Regulation (EC) No. 1334/2008 including EFSAs’ opinion	JECFA	IARC	CLP hazard statement
Menthol^a^	89-78-1	41	Auth.	ADI: 4	–	–
Carvone^a^	99-49-0	38	Auth.	D-Carvone ADI: 1	–	H317
α-Terpineol^a^	98-55-5	28	Auth.	N. c.	–	–
Linalool^a^	78-70-6	27	Auth.	ADI: 0.5 (1979)	–	H317
Tris(2-butoxyethyl) phosphate^a^	78-51-3	24	X	–	–	–
Caryophyllene^a^	87-44-5	24	Auth.	N. c.	–	–
Limonene^a^	138-86-3	21	Auth.	–	–	H317
Eucalyptol^a^	470-82-6	21	Auth.	N. c.	–	–
Menthone^a^	89-80-5	21	Auth.^b^	N. c.	–	–
Piperitone^a^	89-81-6	20	Auth.	N. c.		–
Benzyl alcohol^a^	100-51-6	19	Auth.; ADI: 4 (EFSA Panel on Food Additives and Flavourings (FAF) et al. 2019)	ADI: 5	–	–
Pulegone^a^	15932-80-6	19	x	N. c.	–	–
Butyl palmitate^a^	111-06-8	18	Auth.	–	–	–
Isopulegol^a^	89-79-2	17	Auth.^b^;	N. c.	–	–
Isomenthyl acetate	20777-45-1	15	x	–	–	–
Terpinen-4-ol^a^	562-74-3	15	Auth.	N. c.	–	–
β-Terpineol	138-87-4	15	Auth.	–	–	–
Sorbic acid^a^	110-44-1	12	Auth.;	N. c.. (2003); ADI: 25	–	–
Isomenthol^a^	3623-52-7	11	x	N. c.	–	–
Dihydrocarvone	7764-50-3	11	Auth.	N. c.	–	–
β-Bourbonene	5208-59-3	10	Auth.^b^	N. c.	–	–
Menthyl acetate^a^	16,409-45-3	10	Auth.	N. c.	–	–
Benzyl acetate^a^	140-11-4	9	Auth.	ADI: 5 (1996)	3	–
Neoisomenthol	491-02-1	9	x	–	–	–
Benzaldehyde^a^	100-52-7	8	Auth.	ADI: 5 (2001)	–	–
Anethole^a^	104-46-1	8	Auth.	–	–	–
Geranyl acetate^a^	105-87-3	8	Auth.	ADI: 0.5 (1979)	–	–
Neomenthol^a^	3623-51-6	8	D-Neomenthol (CAS 2216-52-6): Auth.	–	–	–
n-Hexadecanoic acid	57-10-3	8	Auth.	N. c.	-	-
β-Ionone	14901-07-6	8	Auth.	ADI: 0.1 (1984)	–	–
Isomenthone^a^	491-07-6	7	Auth.	N. c.	–	–
Benzoic acid^a^	65-85-0	7	Auth.	ADI: 5 (2001)	–	H372 (lungs) (Inhalation
α-Ionone^a^	127-41-3	6	Auth.^b^	ADI: 0.1 (1984)	–	–
2-Heptadecanone	2922-51-2	6	Auth.	–	–	–
Raspberry ketone^a^	5471-51-2	6	Auth.	N. c.	–	–
Bornyl acetate^a^	76-49-3	6	Auth.	N. c.	–	–
γ-Terpinene^a^	99-85-4	6	Auth.	N. c.	–	–
Cinnamaldehyde^a^	104-55-2	5	Auth.	N. c.	–	–
Caryophyllene oxide^a^	1139-30-6	5	Auth.^b^	N. c.	–	–
p-Anisaldehyde^a^	123-11-5	5	Auth.	N. c.	–	–
Ethyl maltol^a^	4940-11-8	5	Auth.	ADI: 2 (2005)	–	–
Citral^a^	5392-40-5	5	Auth.	ADI: 0.5 (1979)	–	H317
Ethyl salicylate^a^	118-61-6	4	Auth.	N. c.	–	–
Vanillin^a^	121-33-5	4	Auth.	N. c.	–	–
β-Myrcene^a^	123-35-3	4	Auth.^b^	N. c.	2B	–
Butyl stearate	123-95-5	4	Auth.	N. c.	–	–
β-Pinene^a^	127-91-3	4	Auth.	N. c.	–	–
Benzaldehyde propylene glycol acetate	2568-25-4	4	Auth.	N. c.	–	–
cis-β-Farnesene	28973-97-9	4	x	–	–	–
Jasmone	488-10-8	4	Auth.^b^	N. c.	–	–
3-Octanol	589-98-0	4	Auth.	N. c.	–	–
Humulene	6753-98-6	4	x	–	–	–
α-Terpinene^a^	99-86-5	4	Auth.	N. c.	–	–
Geraniol	106-24-1	3	Auth.	N. c.	–	H317
Isoamyl butyrate	106-27-4	3	Auth.	ADI: 3 (1996)	–	–
Maltol	118-71-8	3	Auth.^b^	N. c.	–	–
Carveol	99-48-9	3	Auth.	–	–	–
Piperonal	120-57-0	3	Auth.	ADI: 2.5 (2001)	–	–
Butylated hydroxytoluene^a^	128-37-0	3	ADI: 0.25 (EFSA Panel on Food Additives and Nutrient Sources added to Food (ANS) 2012)	ADI: 0.3 (1995)	3	–
Neryl acetate	141-12-8	3	Auth.	N. c.	–	–
cis-Carvon oxide	18383-49-8	3	x	–	–	–
2-Pentadecanone	2345-28-0	3	Auth.	N. c.	–	–
Hexahydrofarnesyl acetone	502-69-2	3	Auth.	–	–	–
Myosmine^a^	532-12-7	3	x	–	–	–
Ledol	577-27-5	3	x	–	–	–
1-Terpinenol	586-82-3	3	Auth.	N. c.	–	–
Linalyl anthranilate	7149-26-0	3	Auth.	N. c.	–	–
Carvyl acetate	97-42-7	3	Auth.	–	–	–
Eugenol^a^	97-53-0	3	Auth.	ADI: 2.5 (2005)	3	–
p-Cymene^a^	99-87-6	3	Auth.	N. c.	–	–
2-Ethyl-1-hexanol^a^	104-76-7	2	Auth.	ADI: 0.5 (1997)	–	–
β-Citronellol^a^	106-22-9	2	Auth.	ADI: 0.5 (1979)	–	–
Methyl salicylate^a^	119-36-8	2	Auth.	ADI: 0.5 (2001)	–	–
Benzyl benzoate^a^	120-51-4	2	Auth.	ADI: 5 (2001)	–	–
Allyl hexanoate	123-68-2	2	Auth.^b^	ADI: 0.13 (1996)	–	–
Methyl anthranilate	134-20-3	2	Auth.	ADI: 1.5 (2005)	–	–
Caffeine^a^	58-08-2	2	Authorized for the use in certain categories; Safety threshold: 5.7 mg/kg bw/day for adults and 3 mg/kg bw/day for children, adolescents, pregnant and lactating women (EFSA Panel on Food Contact Materials et al. 2017a)	–	3	–
Saccharin	81-07-2	2	x	ADI: 5 (1993)	3	–
Isoeugenol^a^	97-54-1	2	Auth.	N. c.	–	H317
Benzophenone^a^	119-61-9	1	Auth.; TDI = 0.03 (EFSA Panel on Food Contact Materials et al. 2017b)	N. c.	2B	–
Heliotropin	120-57-0	1	Auth.	ADI: 2.5 (2001)	–	–
Estragole	140-46-1	1	x	No ADI allocated (1981)	–	–
Salicylic acid^a^	69-72-7	1	Auth.	N. c.	–	H361d
Triethyl citrate	77-93-0	1	Auth.	ADI: 20	–	–
Methyl eugenol	93-15-2	1	x	No ADI allocated (1981)	2B	–
1-Phenylethanol	98-85-1	1	Auth.	ADI: 0.1	–	–

The table provides information on the status of authorization as food flavoring according to Annex I to Regulation (EC) No. 1334/2008 including EFSA’s opinion, ADIs allocated by JECFA, IARC classification, and CLP hazard statements where relevant. Only compounds with either three or more hits or a toxicological remark are presented. ADI or TDI is expressed in mg/kg body weight

Auth: authorized for use in all categories (Annex I to Regulation (EC) No. 1334/2008); N. c.: No concern at levels of intake (JECFA); ADI: acceptable daily intake (mg/kg bodyweight); TDI: tolerable daily intake (mg/kg bodyweight)

IARC 2B: possibly carcinogenic to humans

IARC 3: unclassifiable as to carcinogenicity in humans

CLP H317—may cause an allergic skin reaction

CLP H361d—suspected of damaging the unborn child

CLP H372—causes damage to organs through prolonged or repeated exposure

x: no entry in the database, meaning no authorization for use as food flavoring in the EU

^a^Included in the in-house library: identification based on spectra and retention times

^b^The database on Food Flavorings (https://ec.europa.eu/food/food-feed-portal/screen/food-flavourings/search) states that the evaluation needs to be completed by the authority

**Table 4:** Calculated limits of substances per pouch in mg based on the respective ADI levels as assigned by EFSA or JECFA

ADI (mg/kg bw/day)	“Average scenario” (5 pouches per day)—limits of substance per pouch (mg)	“Worst-case scenario” (20 pouches per day)—limits of substance per pouch (mg)	Substances concerned
0.1	0.7	0.18	1-Phenylethanol
0.13	0.91	0.23	Allyl hexanoate
0.25	1.75	0.44	Butylated hydroxytoluene (EFSA)
0.3	2.1	0.53	Butylated hydroxytoluene (JECFA)
0.5	3.5	0.88	ADI for the group of citral, geranyl acetate, citronellol, linalool, linalyl acetate, expressed as citral; 2-ethyl-1-hexanol; methyl salicylate
1	7	1.75	d-Carvone
1.5	10.5	2.63	Methyl anthranilate
2	14	3.5	Ethyl maltol
2.5	17.5	4.38	Piperonal; eugenol; heliotropin
3	21	5.25	Isoamyl butyrate
4	28	7	Menthol; benzyl alcohol (EFSA)
5	35	8.75	Benzyl alcohol (JECFA); ADI for the group of benzoic acid, benzoate salts, benzaldehyde, benzyl acetate, benzyl alcohol and benzyl benzoate, expressed as benzoic acid equivalents; ADI for the group of saccharin and its calcium, potassium and sodium salts
20	140	35	Triethyl citrate
25	175	43.75	ADI for the sum of sorbic acid and calcium, potassium and sodium sorbates (expressed as sorbic acid)

For calculation, a consumer of 70 kg bw was assumed to use either 5 nicotine pouches per day (“average consumption scenario”) or 20 pouches per day (“worst case consumption scenario”). To take other exposure sources into account, it was calculated with 50% of the ADI values

## Discussion

Oral nicotine pouches were recently introduced to the market as a new product category (Delnevo et al. 2021). Since these products are not yet object of a particular kind of regulation, publicly available information on the composition and possible health effects is scarce. Therefore, this study aimed to gather information on the general ingredients and aroma substances used in nicotine pouches to shed light onto potential hazards. Packages of 50 different products, 48 nicotine pouches and 2 nicotine-free pouches, were examined for the ingredients listed. To prepare an opinion about these products and, prior to drafting any recommendation toward potential regulation, it is necessary to know much more about the ingredients and flavorings. As described previously, nicotine pouches are based on a filler, mainly cellulose (Robichaud et al. 2020). This is in agreement with our findings. Most abundant technological additives were humectants, mainly propylene glycol, and acidity regulators, primarily sodium carbonate. In a previous study, the pH values of the aqueous extracts of 46 out of the herein assessed 50 pouch samples have been analyzed (Mallock et al. 2022). The pH values ranged from 5.5 to 10.5 with a median pH value of 8.8. All but one sample have led to an alkaline pH of the aqueous extract (Mallock et al. 2022). As nicotine is an alkaline alkaloid, it converts into its unprotonated form at pH values beyond 8. This free-base nicotine is taken up via the oral mucosa more rapidly when compared to the protonated form (Pickworth et al. 2014; Tomar and Henningfield 1997). Acidity regulators can further influence the speed of nicotine uptake, which is considered to be a crucial factor for addiction (de Wit et al. 1992; Henningfield and Keenan 1993). In consequence, these additives may altogether enhance product’s addictiveness. Conversely, if oral nicotine pouches only contained a filler, nicotine, and technological additives, they would elicit an unpleasant bitter taste or even oral irritations and pain (Carstens and Carstens 2022). It can be assumed that nicotine pouches are marketed with various flavor supplements to circumvent this discomfort. In this study, 21 different flavor categories according to Krüsemann et al. (2019) were identified and about half of all flavor descriptions indicated the use of mint or a cooling agent. It should be noted that the sampling process was not aimed at identifying all available flavor categories. Thus, this number only reflects a fraction of what can be expected to be present on the market. Sweeteners and aroma additives were declared on almost all products. Mostly used sweeteners were xylitol, acesulfame K, and sucralose. Aroma additives used remained mainly undefined. Preservatives were listed on 17 product packages, although the exact identities of these were not specified on six of them. As some preservatives, e.g., sorbic acid, are known to potentially induce hypersensitivity, a clear declaration should be available. To identify flavorings and other substances contained in the pouches, a screening approach using LLE and GC/MS was applied.

186 substances were identified, some of which with toxicological concern according to IARC or CLP regulation. However, this does not automatically equal ‘risk by product use’, as the factor ‘exposure’ has to be taken into account. Thus, toxicological thresholds and safety limits should be considered. Authorities and organizations such as EFSA and JECFA have defined ADI values for many substances. The daily intake of these substances below their ADI limits is considered safe when all possible intake sources are included.

Possible adverse effects from pouch use include systemic toxic effects through ingestion, and local adverse effects such as oral lesions. Oral acute toxicity of the products most likely originate from the compound nicotine, which is added at alarmingly high concentrations to some products. Based on the information extracted from patents belonging to Phillip Morris, flavors are added to the pouches in amounts of 0.01–15% by weight of the pouch (Bruun 2018; Stahl et al. 2020). In the study by Mallock et al. (2022), the median pouch weight was determined to be 0.643 g with a minimum weight of 0.305 g and a maximum weight of 1.246 g. Assuming that 1% of flavor was added to a pouch of 0.64 g, this would result in a total exposure to 6.4 mg flavors per pouch. The calculated intake limits were based on a 50% exhaustion of the ADI, as further exposure sources through diet or cosmetic occur as well. Yet, the ADI values would be exceeded for all substances with an ADI of up to 1 mg/kg bw or 4 mg/kg bw when 5 pouches or 20 pouches were consumed per day, respectively, without any further dietary sources taken into account. Therefore, adverse effects cannot be ruled out to be induced by these substances. Further, the products may be applied at the same part of the gum at every use. Local concentrations might be significantly higher than after ingestion and adverse effects at the site of use are thus most likely. In fact, mucosal changes have been reported in users of snus and nicotine pouches (Miluna et al. 2022). Shao et al. (2022) analyzed posts related to nicotine pouches on the platform Reddit and found that many users experienced gum problems upon usage of nicotine pouches. Whether gum problems referred to oral mucosa defects could turn into malignant oral lesions after a prolonged use of nicotine pouches remains to be studied. Although the above-mentioned fragrance allergens are authorized in food, it is conceivable that ADI values for citral, linalool, and carvone are getting exceeded depending on the particular product and the use behavior. The ADI for an entire compound group describes the sum of the acceptable intake for all of the group members combined, meaning an ADI could already be exhausted below the intake limit of a certain compound as substances of the same group could be mixed together. Further, the TDI of benzophenone (0.03 mg/kg bw) could easily be exceeded even if low amounts were used in nicotine pouches.

Local toxicity of nicotine pouches should be studied in biological settings. Therefore, as a second part of this study which is described in another paper, the in vitro toxicities of five nicotine pouch extracts were assessed in HGF-1 cells (Rinaldi et al. 2023, submitted). It has been demonstrated that some nicotine pouch extracts were cytotoxic and increased the expression levels of genes related to oxidative stress (*HMOX1*) and inflammation (*IL6*). The production of intracellular reactive oxygen species was mainly observed for the CRP1.1 snus product but not for nicotine pouch extracts. Since the detected effects were mostly not attributable to the nicotine content, the contribution of flavors to this toxic marker seems reasonable (Rinaldi et al. 2023, submitted). This is supported by the findings of Shaikh et al. (2022) who showed that flavored nicotine pouches showed higher cytotoxicity when compared to tobacco flavored snus in human gingival epithelial cells (HGEP) (Shaikh et al. 2022).

Besides their contribution to toxicity, flavorings and other additives can increase the product attractiveness for nicotine-naïve individuals that are not adapted to the harsh sensation of oral nicotine exposure. Further, the use of certain ingredients can be accompanied with positive health expectations. On the two nicotine-free products, caffeine-containing herbs were declared that may create the expectation that these products will keep the consumer awake. In addition, both investigated products contained ashwagandha, a plant that has been used as a herbal remedy to treat stress-related disorders. This could convey an expression of a healthy product that can be used in stressful situations. On the other hand, ashwagandha has been suspected to be responsible for liver injury. Certain ingredients of ashwagandha are being detoxified by glutathione *S*-transferases and its cofactor GSH at normal doses. At very high doses, however, this system could be overwhelmed, thus leading to DNA damage and liver injury (Siddiqui et al. 2021). Therefore, additives to nicotine-free products should be regulated and monitored the same way as warranted for nicotine-containing pouches. Nicotine-free pouches, as a seemingly “risk-free product”, might be attractive for young people or never nicotine users that could thereby get used to the sensation of oral pouches, potentially causing use of nicotine pouches in the long run.

In summary, this study has revealed some concerns regarding substances that were present in nicotine pouches: It is likely that ADI levels of some flavorings are exceeded even through moderate consumption of nicotine pouches, possibly resulting in adverse effects. This should be followed up with a quantitative assessment. Impurities, e.g., the minor tobacco alkaloid myosmine, were detected, indicating that the used ingredients were not always of sufficient purity. Substances were identified that may cause an allergic skin reaction according to the harmonized hazard classification by CLP. Although all of them are authorized as food flavorings, it remains unclear whether potential sensitization is fostered due to the repeated application at the same local site. Substances were detected that are classified as “possibly carcinogenic” by IARC with one of them, methyl eugenol, not being authorized as food flavoring. Two pouches were tested that did not contain nicotine but other pharmacologically active ingredients such as ashwagandha extract or caffeine.

Based on these results, conclusions for a future regulation of ingredients in oral (nicotine) pouches can be derived. The regulation should not only consider nicotine-containing pouches but also apply to nicotine-free variants. First, for safety reasons, ingredients used should be authorized for its use in food and should fulfill the requirements by food law. Likewise, the pouch material should comply with regulations for food contact materials. In addition, it is warranted that some requirements specified for tobacco products are applied. At least, provisions in Art. 7(6) points (a) and (b) of the Tobacco Products Directive (European Parliament and the Council of the European Union 2014) should be considered for the adaption of oral pouches, thereby banning “vitamins or other additives that create the impression that a tobacco product has a health benefit or presents reduced health risks” and “caffeine or taurine or other additives and stimulant compounds that are associated with energy and vitality”. Further, analogous to requirements for nicotine-containing liquids for e-cigarettes, only ingredients of high purity should be used in the manufacture of oral (nicotine) pouches.

## Limitations and outlook

The aim of the present study was to gain first insights into the composition of oral pouches using a screening approach. For this, a convenience sampling method was used. No exhaustive market analysis was performed and the abundance of identified compounds is not necessarily representative for the entire market. Only substances were identified that were accessible with the analytical methods used, meaning that substances have to be stably extractable under applied LLE conditions, they have to be GC-amenable and they have to be included in the consulted databases. Thus, the list of substances most likely is not exhaustive. It should be noted that no information on the stereochemistry was obtained with the used separation method and that only the free acid form was detected for salts of organic acids with no identification of the counter ion. Different stereoisomers or salt forms usually have different CAS numbers and may differ in their toxicological properties. It was not feasible to carry out a comprehensive hazard assessment for all identified substances nor to perform a quantitative analysis. However, the presented work should be understood as a starting point for further assessments. For example, for substances for which the ADI values are likely exceeded, the quantification of the exposure would enable a more precise risk assessment. Furthermore, this work has focused on the contribution of flavorings to product toxicity. The influence on abuse liability and that on product attractiveness toward vulnerable groups need to be addressed in future studies.

## Supplementary Information

Below is the link to the electronic supplementary material.

Supplementary file1 (XLSX 45 KB)

## Data Availability

All data generated or analysed during this study are included in this published article and its supplementary information files.
